# Effectiveness of decontamination protocols when analyzing ancient DNA preserved in dental calculus

**DOI:** 10.1038/s41598-021-86100-w

**Published:** 2021-04-02

**Authors:** Andrew G. Farrer, Sterling L. Wright, Emily Skelly, Raphael Eisenhofer, Keith Dobney, Laura S. Weyrich

**Affiliations:** 1 Australian Centre for Ancient DNA, School of Biological Sciences, University of Adelaide, Adelaide, South Australia Australia; 2 The Department of Anthropology, The Pennsylvania State University, University Park, PA USA; 3 Department of Archaeology, University of Sydney, Sydney, NSW Australia; 4 Australian Research Council Centre of Excellence for Australian Biodiversity and Heritage, University of Adelaide, Adelaide, South Australia Australia; 5 The Huck Institute of Life Sciences, The Pennsylvania State University, University Park, PA USA

**Keywords:** Microbial communities, Environmental microbiology, Microbial ecology

## Abstract

Ancient DNA analysis of human oral microbial communities within calcified dental plaque (calculus) has revealed key insights into human health, paleodemography, and cultural behaviors. However, contamination imposes a major concern for paleomicrobiological samples due to their low endogenous DNA content and exposure to environmental sources, calling into question some published results. Decontamination protocols (*e.g.* an ethylenediaminetetraacetic acid (EDTA) pre-digestion or ultraviolet radiation (UV) and 5% sodium hypochlorite immersion treatments) aim to minimize the exogenous content of the outer surface of ancient calculus samples prior to DNA extraction. While these protocols are widely used, no one has systematically compared them in ancient dental calculus. Here, we compare untreated dental calculus samples to samples from the same site treated with four previously published decontamination protocols: a UV only treatment; a 5% sodium hypochlorite immersion treatment; a pre-digestion in EDTA treatment; and a combined UV irradiation and 5% sodium hypochlorite immersion treatment. We examine their efficacy in ancient oral microbiota recovery by applying 16S rRNA gene amplicon and shotgun sequencing, identifying ancient oral microbiota, as well as soil and skin contaminant species. Overall, the EDTA pre-digestion and a combined UV irradiation and 5% sodium hypochlorite immersion treatment were both effective at reducing the proportion of environmental taxa and increasing oral taxa in comparison to untreated samples. This research highlights the importance of using decontamination procedures during ancient DNA analysis of dental calculus to reduce contaminant DNA.

## Introduction

Microbial communities within the human microbiota vary across different body sites (*e.g.,* the gut, skin, and oral cavity). The human body relies on resident microbial communities because they perform an array of essential functions, such as release inaccessible nutrients from food^1^, remove dead epithelial cells from the skin^2^, and facilitate tooth enamel remineralization^3^. These diverse communities are intricately linked with the human immune and endocrine systems^4^, and microbiota alterations have now been linked to a wide range of diseases, including kidney and respiratory conditions^5,6^, oral pathologies^7^, allergies^8^, obesity^9^ and mental disorders^10^. Numerous factors, including diet, age, gender, environment, genetics, disease exposure, and pharmaceutical use^11–13^, are each known to shape the composition of microbiota. As a result, it is possible that shifts in microbiota may provide insights into the past life history of an individual. Examinations of microbiota from temporally, geographically, and culturally diverse populations may glean information about social stratification, cultural or dietary change, and the origins of modern diseases^14^.

Ancient DNA (aDNA) analyses can offer valuable insights into the evolution of these human microbial communities and their response to various cultural and ecological factors over multiple generations. Calcified dental plaque (calculus) consistently enables the reconstruction of ancient human microbiota^15^. Dental calculus is formed by the calcification of the diverse bacterial biofilm that forms on the tooth surface^16^. This calcium matrix preserves and protects the bacterial cells from many of the abiotic and biotic factors that degrade soft tissues post-mortem. Recent studies of ancient dental calculus have revealed changes in oral microbiota that are correlated to alterations in diet and lifestyle, including the implementation of agricultural practices, and an increase in oral pathogens over time^17^. Dental calculus preserves both host and dietary DNA, but more than 99% of the preserved DNA is microbial in origin^18^. It retains more endogenous DNA in ancient samples than other ancient substrates, such as bone and coprolites^19^. However, ancient samples are highly susceptible to contamination introduced from burial, storage, and laboratory environments, all of which can drastically alter microbial composition. Contamination, therefore, poses a significant risk for ancient dental calculus analysis^19–23^, as it is an unwanted source of variation that obscures biological factors of interest. For these reasons, strict aDNA protocols must be followed, including methods that reduce and monitor contaminant DNA contributing to a sample.

Failure to account for contaminant DNA introduces spurious heterogeneity into ancient microbiota data, which can lead to misinterpreted results^24^. For example, microbial DNA found in different manufacturing batches of DNA extraction kits can create signals within data sets that appear to be biological^24^. While many aDNA research teams limit laboratory contamination by working in dedicated aDNA laboratories, the field has yet to standardize other control measures^25^. Although evidence suggests that sequencing extraction and non-template amplification (*i.e.,* PCR negatives) controls can help monitor laboratory contamination^26^, some research teams fail to include such sequencing data in their publications^22^. However, such controls cannot detect contaminant DNA that was present on the sample prior to entering the facility^15^. The surface of ancient samples can contain microbial DNA from a wide range of contaminating sources, such as sediment, storage materials, and handling during and after excavation. Therefore, the field should devote efforts to minimize environmental microbila contaminants prior to DNA extraction and limit the inclusion of such signals using bioinformatic tools.

Most aDNA research involves a decontamination protocol prior to DNA extraction to reduce the amount of environmental DNA in calculus samples^17,18^. The expectation is that these methods reduce environmental signals while increase the DNA yield of ancient oral microbiota, as well as improve bioinformatic filtering of contaminants. However, protocols for decontaminating dental calculus varies among research groups (*e.g.,* Adler et al., 2013; Warinner et al., 2014), making it difficult to trust the comparisons among datasets. While the two widely-used pre-extraction treatments, sodium hypochlorite and ethylenediaminetetraacetic acid (EDTA), improve the recovery of endogenous human DNA from bone^27^, it is uncertain whether these protocols alter the endogenous microbial composition in calculus^28^. To assess the impacts that decontamination treatments have on archaeological dental calculus, we compared untreated samples to four protocols: 1) ultraviolet (UV) irradiation; 2) 5% sodium hypochlorite (NaClO) immersion; 3) pre-digestion in EDTA^18^; and 4) UV irradiation + 5% sodium hypochlorite immersion (UV + NaClO)^17^. Our analysis included 26 samples from a well-preserved, medieval archaeological site (York, UK)^29,30^, each of which have a consistent oral microbial signal^17^. These direct comparisons of aDNA decontamination protocols on ancient dental calculus samples can serve as a resource for the future analysis of ancient oral microbiota.

## Materials and methods

### Archaeological context and site information

Single dental calculus samples were collected from 26 different individuals buried at a Medieval archaeological site in York, UK. Each sample was randomly placed into either the untreated group or one of the four treatment groups described below. This approach was taken because dental calculus samples are heterogenous and often too small to subsample into five separate groups (i.e. average sample size was 10–15 mm^2^). The dates for these dental calculus samples ranged from 1170–1290 CE. This cemetery was excavated in 1983^29^, and archaeological examination of the human remains ended following reburial requests from the Jewish community. However, analyses on the site and skeletons were recorded^30^ along with dental calculus samples which were collected for future investigations. Ethical approval was obtained from the Human Research Ethics Committee of the University of Adelaide; all experimental protocols and analyses were performed and completed under ethical approval to study ancient human dental calculus (University of Adelaide: H-2012–108). All methods were performed in accordance with the guidelines outlined in Cooper and Poinar (2000)^25^. The population consisted of individuals with middle-to-poor socio-economic standing, with 98.1% buried in single graves. Dental caries was observed in 59.5% of individuals, and periodontal disease was present in > 80%^29^. Calculus was sampled on-site at the time of excavation and stored in glass vials until transport. Samples were transported to the aDNA facility based in the Australian Centre for Ancient DNA (ACAD), Adelaide, Australia for processing. Previous aDNA analysis of calculus using the UV and sodium hypochlorite treatment from this site has revealed that oral microbial communities were not statistically different between samples and were distinct from other cultures (e.g. Neolithic farmers and hunter-gatherers) and archaeological sites (e.g. in Germany and Poland) based on microbial composition alone^17^.

### Decontamination protocols

**Figure 1:**
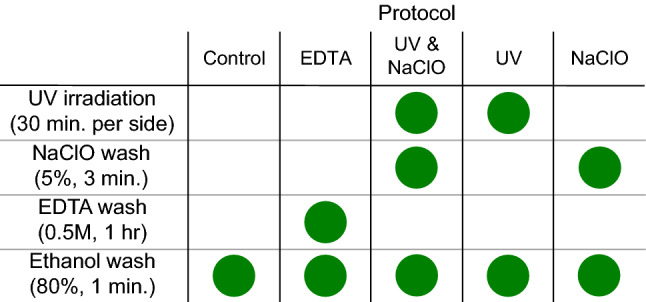
The five decontamination protocols applied. A workflow, from top to bottom, for each of the decontamination protocols applied to the groups of ancient dental calculus samples. Green dots represent use of that treatment on each sample being analysed.

Each of the five groups underwent a different decontamination protocol prior to DNA extraction. The protocols are summarized in Fig. 1 and were as follows: untreated controls (n = 5);^17^; UV treatment (n = 5); NaClO treatment (n = 6), UV + NaClO treatment (n = 5); and, EDTA treatment (n = 5)^18^. The UV + NaClO treatment exposed dental calculus fragments to UV radiation for 30 min on each side, followed by a submersion in 3 mL of 5% sodium hypochlorite in a sterile petri dish for 3 min^17^. The individual UV-only and NaClO-only treatments used the respective element of the UV + NaClO protocol. For the EDTA treatment, calculus fragments were submerged in 1 mL 0.5 M EDTA for 1 h^18^. Following the decontamination protocols, all samples were washed in 1 mL of sterile 80% ethanol for one minute to remove residual chemicals (*i.e.,* EDTA or NaClO) prior to extraction. The ethanol washes from each sample (n = 26), as well as control ethanol samples (n = 3), were evaporated, and the resulting DNA was suspended in TLE buffer (500 μl Tris HCL (1 M), 10 μl EDTA (0.5 M), and 50 ml dH_2_O)^31^.

### DNA extraction

All samples, except for the ethanol washes, underwent an in-house, silica-based DNA extraction, as previously described^32^. To account for small sample sizes, total volumes of lysis and guanidinium DNA binding buffer were reduced as follows: 1.8 mL lysis buffer (1.6 mL 0.5 M EDTA (0.5 M), 200 μL SDS (10%), and 20 μL proteinase K (20 mg/ml)) and 3 mL guanidinium DNA binding buffer. Two extraction blank controls were included for every seven samples for the amplicon process, while one was performed for shotgun.

### 16S rRNA amplicon approach

A 289 base pair stretch from the V4 region of the 16S ribosomal RNA (rRNA) encoding gene (position 515–806 of the *E. coli* reference genome) was amplified in triplicate from all samples (dental calculus, resuspended ethanol washes, and extraction blank controls) alongside an additional PCR negative control using a universal forward primer and sample specific, barcoded reverse primer^33^. This amplicon was previously targeted in the study of ancient dental calculus by Adler et al. (2013) and is used to obtain sufficient resolution of bacterial taxonomy to allow analysis of the microbial community^17^. Each PCR reaction contained: 17.5 μL sterile H_2_0, 1 μL of DNA extract, 0.25 μL of Hi-Fi taq (Life Technologies), 2.5 μL of 10X Hi-Fi reaction buffer, 1 μL MgCl_2_ (25 mM), 0.2 μL dNTPs (10 mM), and 1 μL each of the forward and reverse primers. Samples were amplified using the following conditions: initial denaturing (95 °C, 6 min), followed by 37 cycles of denaturing (95 °C, 30 s), annealing (50 °C, 30 s), and elongation (72 °C, 30 s), and finally adenylisation (60 °C, 10 min). High numbers of cycles can cause inflation of diversity estimates^34^. However, the high cycle number is normal for ancient DNA where low concentrations of input DNA are experienced^17,18^. Following amplification, the triplicate reactions were pooled, and PCR products were visualized by electrophoresis on a 2.5% agarose gel. Samples were quantified (Qubit 2.0, Life Technologies) before being pooled at equimolar concentrations and purified (Ampure, Agencourt Bioscience). The pooled sample (*i.e.,* DNA library) was quantified using the Tapestation and the KAPA SYBR Fast Universal master mix qPCR assay (Geneworks). DNA sequencing was completed using the Illumina MiSeq 150 bp paired end chemistry (Illumina, San Diego, CA,^35^USA) at the Australian Genome Research Facility Ltd (AGRF), Adelaide. Sequencing data can be found in Sequence Read Archive (https://www.ncbi.nlm.nih.gov/sra) under the accession number PRJNA688065.

### 16S rRNA amplicon bioinformatic analyses

Sequences were demultiplexed and quality filtered in QIIME (V1.8)^36^ using the split_libraries_fastq.py script with parameters: barcode error = 0 and quality score > 20. Operational taxonomic unit (OTU) picking was completed against GreenGenes (V13.8)^37^ with 97% similarity using both closed and open reference methods. The closed reference OTU dataset only includes sequences that match references within the GreenGenes database; the open reference dataset also included OTUs without reference matches. To remove contaminant DNA introduced through laboratory processing, OTUs identified in negative controls and as common laboratory contaminants^24^ were removed from dental calculus samples processed in the same batch. Ethanol washes, which were not expected to have a high biomass or necessarily to be representative of human microbiota, were filtered by the OTUs present in the control ethanol samples. Finally, singletons (OTUs present only once) were removed from the data.

Following filtering, bioinformatics analyses for the 16S rRNA amplicon dataset were conducted within QIIME (V1.8). To examine differences in diversity between the different decontamination steps, a variety of analyses were performed. Alpha diversity (observed species) was calculated for each treatment group at rarefaction levels from 0 to 2,000 (in intervals of 10) using closed and open reference datasets in QIIME. A Goodness of fit test (G-test) was applied to detect significant differences in genus-level taxa between untreated samples and each of the decontamination protocols. To reduce false positives generated by rare taxa, OTUs below 0.1% of the total taxa present were removed before performing the G-test. To identify the environmental taxa impacted by the decontamination protocol, genus-level taxa that were significantly different (p < 0.001) were classified as environmental or oral based on their presence or absence (respectively) in the Human Oral Microbiome Database (HOMD) (homd.org).

Several statistical assessments were performed to identify taxa that were significantly altered by the different treatments. First, a one-way ANOVA was applied to test if the average frequency of OTUs in each protocol group had altered in relation to the untreated group. Next, for each sample, OTUs identified in the G-test analysis were ranked as increasing or decreasing relative to the untreated proportion. A one-way ANOVA was performed to identify taxa that significantly differed between the four treatment groups. Finally, taxa released into the ethanol washes were classified as environmental or oral using HOMD, and the ratio of oral to environmental taxa was assessed.

### 16S rRNA amplicon source tracker analysis

SourceTracker (V0.9.6)^50^ was used to identify the proportions of endogenous and contaminant signal in each sample. SourceTracker differentiates the community profiles in the ‘source’ to those of the ‘sink’ (*i.e.* the sample), using Bayesian methods to associate the extent of contribution of each source to a sink. Comparative data included dental calculus from modern (n = 6) and Industrial Revolution (n = 3) individuals accessed from the Online Ancient Genome Repository (OAGR)^51^. Additionally, preprocessed 16S rRNA gene datasets were downloaded from the Qiita database for comparison (qiita.microbio.me) and included: human skin samples (n = 11)^38^ and environmental samples from agricultural soil (n = 8), temperate soil (n = 4), forest soil (n = 4), tropical soil (n = 5), and park soil (n = 6) (Study IDs: 232, 808, 846, and 1674. qiita.microbio.me). Specifically, a subset of skin samples was used to reduce bias from unevenly sized reference groups (samples: P15024, P15268, P15733, P16107, P16187, P16199, P16304, P16320, P16393, P16399, and P16562 were used). SourceTracker was run with default parameters (1,000 subsampling, 10 iterations per sink sample) in R version 3.1.0.^39^ using the QIIME wrapper. The suitability of the source populations was confirmed using the “take-one-out” method, which demonstrated that the samples within each reference group were more similar to one another than samples in any other group.

### Shotgun metagenomic library preparation and sequencing

Metagenomic shotgun libraries were constructed as previously described^35^ without the enzymatic damage repair, as used by other ancient dental calculus studies^40^. Briefly, 20 μL of DNA extract underwent enzymatic polishing to produce blunt ended fragments, before the ligation of truncated 7-bp forward and reverse uniquely barcoded Illumina adaptors, and finishing by filling in the gaps between the adaptor sequences and the DNA sequence. MinElute Reaction clean-ups (Qiagen) were completed after both enzymatic polishing and barcode ligation steps. Libraries were amplified in triplicate by PCR for 13 cycles with Illumina amplification primers^35^. Each PCR reaction contained: 13.25 μL sterile H_2_0, 5 μL of library DNA, 0.25 μL of Hi-Fi taq (Life Technologies), 2.5 μL of 10X Hi-Fi buffer, 1.25 μL MgSO_4_ (50 mM), 0.25 μL dNTPs (100 mM), and 1.25 μL each of the forward and reverse primers. Cycling conditions were as follows: 94 °C for 12 min; 13 cycles of 94 °C for 30 s, 60 °C for 30 s, 72 °C for 40 s (increasing increments of 2 s per cycle); and ending at 72 °C for 10 min. PCR products were pooled and cleaned with AxyPrep magnetic beads (Axygen Scientific, Inc.), and then re-amplified with GAII Indexed Illumina primers^35^, using the above cycling conditions and a modified PCR reaction: 12.75 μL sterile H_2_0, 2 μL of purified Library DNA, 0.25 μL of AmpliTaq Gold (Life Technologies), 2.5 μL of 10X Gold buffer, 2.5 μL MgCl_2_ (25 mM), 0.625 μL dNTPs (10 mM), 1.25 μL Illumina amplification primer, and 1.25 μL GAII Ilumina indexed adaptor. Libraries were purified again prior to quantification using TapeStation (Aligent) and pooled to a final 4 nmol/L DNA concentration before being sequenced with an Illumina NextSeq 500, mid output 300 cycle (2 x 150 bp) kit (Illumina, San Diego, CA, USA) at the Australian Genome Research Facility Ltd. (AGRF), Adelaide.

### Shotgun metagenomics bioinformatic and statistical analyses

Shotgun metagenomic sequencing data was converted into FASTQ format using the free Illumina bcl2fastq (version 2.15) software (https://support.illumina.com/sequencing/sequencing_software/bcl2fastq-conversion-software.html) before being trimmed, collapsed by overlapping paired sequences, and demultiplexed by unique combinations of P5/P7 barcodes using AdapterRemoval2^41^. Collapsed reads were then aligned against a reference database containing 47,713 bacterial and archaeal RefSeq genome assemblies^42^ using MALT v 0.3.8^43,44^ with default settings. The resulting blast-text files were converted into RMA files via the blast2rma script^45^ with the following LCA (Lowest Common Ancestor) parameters: –minScore = 42; –maxExpected = 0.01; –minSupportPercent 0.1; –lcaAlgorithm = weighted; –lcaCoveragePercent = 80. Data was compared in MEGAN6CE (version 5.11.3)^45^. A biom table was exported from MEGAN6CE into QIIME2 (v. 2019.10)^46^. Species identified in EBCs were completely removed from the taxonomic table using the ‘qiime feature-table filter-samples’ quality control plug-in^47^.

A feature table was generated using the ‘qiime feature-table summarize’ plugin in QIIME2 (v. 2019.10). Microbial alpha and beta diversities were also calculated and analyzed using the ‘qiime diversity alpha-group-signfiance’ and ‘qiime diversity beta-group-significance’ plugins. For these analyses, OTUs observed in the extraction blank were removed using the ‘qiime feature-table filter-samples’ plugin. Next, samples were rarefied to 70, 601 (the lowest amount of high-quality reads observed in a sample; i.e., sample ID 8833). Alpha (Shannon’s diversity and Simpson’s index) and beta ( Bray–Curtis dissimilarity) diversity differences among groups were tested using Kruskal–Wallis analysis of variance. Ordination of Bray–Curtis distances was done using principal coordinate analysis (PCoA) and visualized using Emperor in QIIME2^48^. In addition, we performed a G-test to explore differences in OTU abundance among the different sample groups using the group_signficance.py script in QIIME and the Bonferroni method to correct for multiple comparisons. The Kruskal–Wallis test for all the comparisons were statistically insignificant with p-values greater than 0.05.

### Shotgun metagenomics source tracker analysis

For the shotgun analysis, “source” contributions to samples were predicted from rarefied species-level bacterial and archaeal taxonomic frequency tables from MALT using SourceTracker (V0.9.8)^49^. Comparative data included gut (n = 19)^50–52^, plaque (n = 10)^53^, saliva (n = 10)^54,55^, skin (n = 6)^56,57^, and soil samples (n = 6)^58,59^ (Supplemental Information). The raw shotgun sequences for these source communities were downloaded and processed in the same fashion as the samples in the current study. A species-level table, filtered of taxa matching those identified in the EBCs, was used as input. SourceTracker was ran using the default settings, except with a rarefaction depth of 55,833—the lowest number of species per sample in the biom table. The predicted percentage contributions of each potential source to the sinks were visualized using the following R packages: *ggplot2*^60^*, dplyr*^61^*, tidyr*^62^*,* and *reshape2*^63^*.*

## Results

### 16S rRNA amplicon analyses

#### Reduction in detectable contaminant OTUs

**Figure 2:**
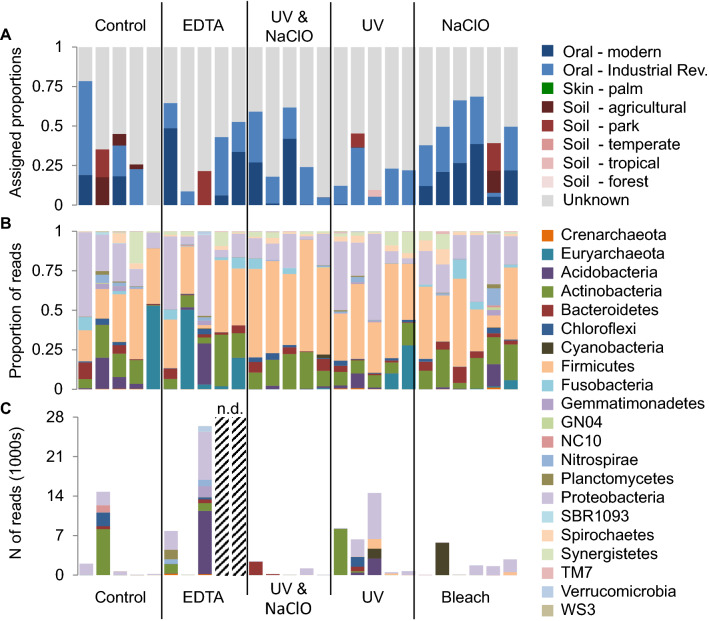
Contamination and taxonomic profiles of each sample based on 16S rRNA amplicon sequencing, grouped by decontamination protocol. The proportion of oral, skin, soil, and unknown OTUs in each sample. Proportions were defined by comparison to reference samples of oral, skin and soil microbial communities using the Bayesian modelling program SourceTracker (**A**). The OTUs identified within each phylum are displayed for each calculus sample (> 0.08% of total proportion) (**B**) and for each ethanol wash (**C**). These analyses used closed reference OTUs (*i.e.*, OTUs identified in the GreenGenes database).

Our first aim was to assess known contaminant OTUs using an amplicon approach, as this sequencing technique can detect rare taxa and low-level contaminants more frequently than shotgun metagenomic sequencing. The SourceTracker analysis was applied to the 16S data to predict the amount of environmental DNA in each sample (Fig. 2A). Samples in the untreated group were, on average, comprised of 9.1% soil OTUs. In contrast, the EDTA treatment and UV + NaClO treatment had 4.3% and < 0.01% of soil OTUs, respectively. Specifically, four out of the five EDTA treated samples had no detectable soil component, while a single EDTA treated sample was comprised of 21.5% soil OTUs. Similarly, only one sample from the UV + NaClO treatment had a detectable soil signal, which represented < 0.01% of the sample. When the UV and NaClO treatments were performed independently, UV treated samples had an average soil component of 6.3%, while NaClO treated samples had 5.3%. We found no evidence of skin microbiota in any sample. Furthermore, samples in the untreated group were comprised of 27.8% oral OTUs. A relative increase in the proportion of oral OTUs was detected in both published treatments (33.8% in the EDTA while 33.6% in the UV + NaClO method), as well as the NaClO treatment (46.7%). Surprisingly, UV treated samples had the lowest average oral component of all the groups (19.8%). Importantly, all decontamination protocols reduced environmental contaminant OTUs, while the EDTA, UV + NaClO, and NaClO treatments were additionally able to increase oral microbiota proportions relative to the untreated samples.

#### Decontamination generally decreases amplicon sample diversity

**Figure 3:**
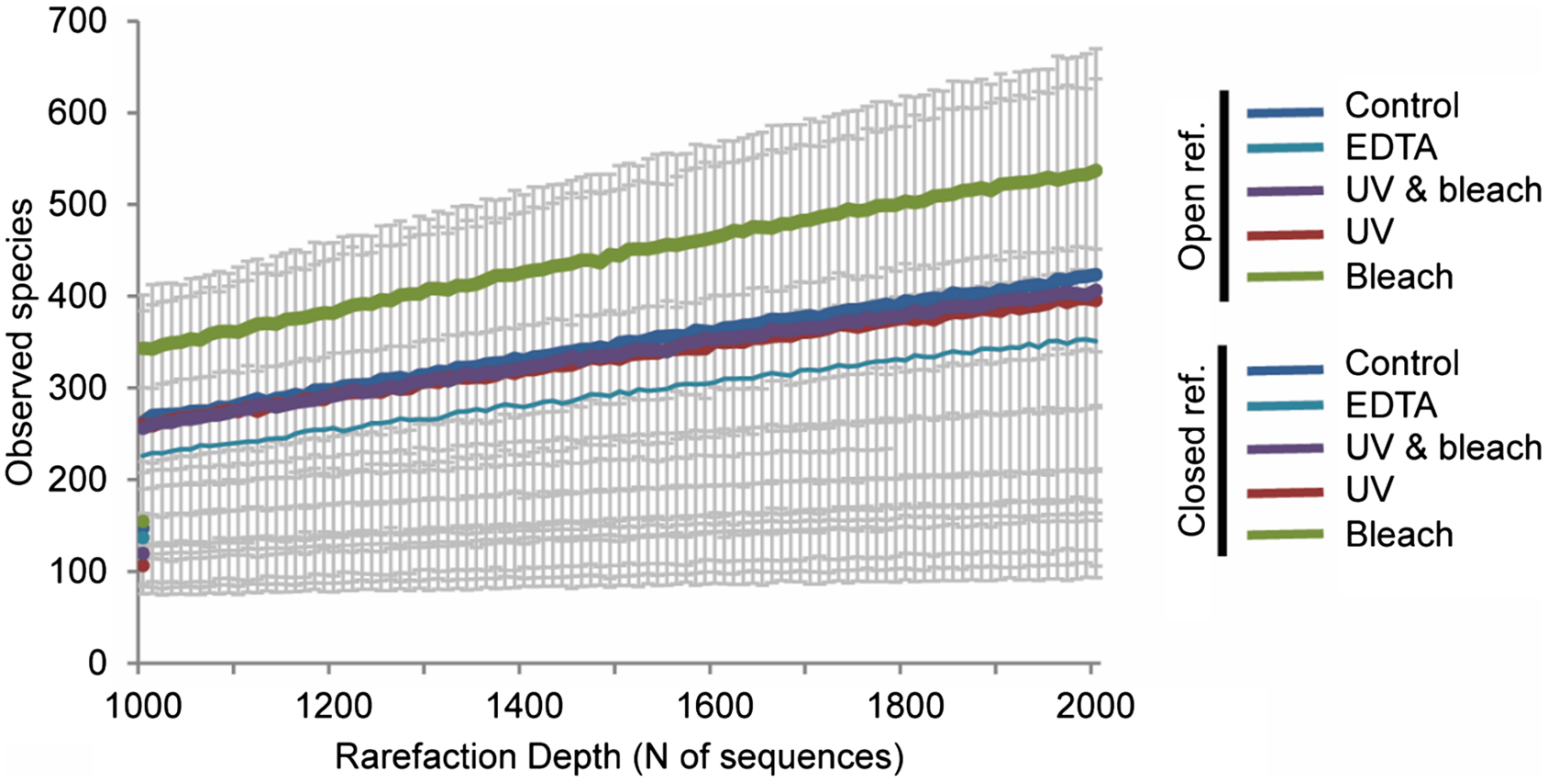
Average alpha diversity within protocols. Lines represent the average alpha diversity (observed species) of all samples processed with the same decontamination protocol. Dotted lines signify the diversity calculated within the closed reference OTUs (OTUs identified in the GreenGenes database) and solid lines represent diversity detected within the open reference OTUs (Closed reference OTUs plus OTUs without matching sequences in the GreenGenes database). The colours represent the different protocols, and the error bars (one standard deviation) are shown in grey.

An effective decontamination method should reduce the numbers and relative abundances of contaminant taxa, and therefore, also reduce the overall diversity within calculus samples. To test this, alpha diversity was examined in both open and closed reference OTU datasets (Fig. 3). The untreated group had the largest variation among samples, resulting in non-significant differences (p > 0.05). However, there were interesting trends across the different treatments. Both the EDTA and UV + NaClO protocols reduced the microbial diversity when compared to the untreated samples. For the closed reference data (where only OTUs matching the GreenGenes database were considered), the average diversity in the EDTA treatment was 7.4% lower than the untreated group. The UV + NaClO treatment had a greater impact and was 18.5% lower than the untreated group. For open reference OTUs (which also include de novo OTUs), the EDTA treatment reduced diversity by 12.6%, while the UV + NaClO treatment reduced diversity by 1.1%. UV treatment also reduced diversity in both closed and open reference datasets (26.4% and 0.9%, respectively). Conversely, the NaClO treatment increased the diversity in both cases. This is particularly notable in the open reference data; when de novo OTUs were included, there was an average 29.4% increase in diversity in the NaClO treated group compared to the untreated group, which suggests that NaClO treatment alone may artificially increase amplicon bacterial diversity. These findings indicate that the environmental DNA is removed from dental calculus during decontamination, while overall diversity is not significantly impacted.

#### Exclusion of environmental amplicon taxa following decontamination

**Figure 4:**
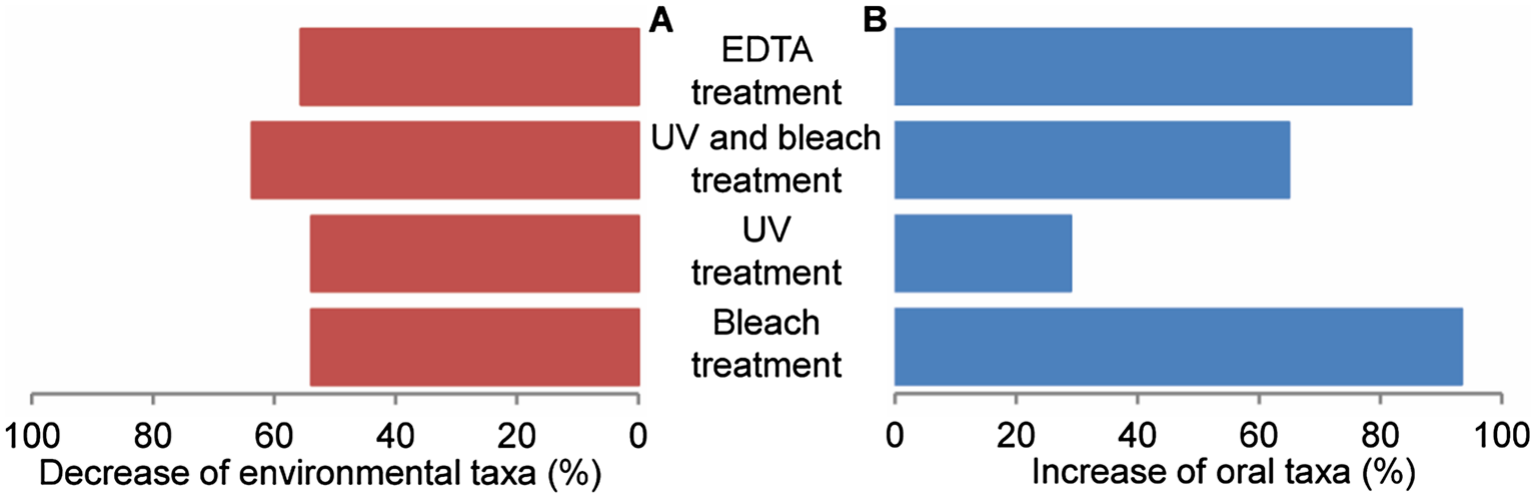
Percentage change in environmental and oral taxa. Taxa with significantly different abundances from the no treatment controls were identified using the G-test. Of these significant taxa, we summarized the percentage of environmental taxa (absent from HOMD) that decreased relative to the no treatment controls (**A**), and the percentage of oral OTUs (present in HOMD) that increased relative to no treatment controls (**B**).

We used a G-test to determine which genera significantly changed in frequency during each treatment relative to the untreated group. *Methanobrevibacter* taxa were excluded from this analysis, as abundance measures based on 16S rRNA gene sequencing are heavily biased for this group^64^. We summarized the percentage of environmental taxa (i.e., absent from the Human Oral Microbiome Database (HOMD)) with significant differences in frequencies (p < 0.001) relative to the no treatment controls (Fig. 4A) and the percentage of oral OTUs (present in HOMD) that increased relative to no treatment controls (Fig. 4B). Of the published protocols, EDTA treatment reduced a smaller proportion of environmental taxa compared to the UV + NaClO protocol (55.6% compared to 63.6%). The percentages of oral taxa were higher in the EDTA (85%) and UV + NaClO protocols (64.8%) than in the untreated samples. In contrast, the UV-only treatment reduced 53.8% of environmental taxa, while increased only 28.9% of oral. NaClO treatment also reduced 53.8% of environmental taxa but promoted the highest proportion of oral taxa (93.3%). However, these differences were non-significant when tested with a one-way ANOVA (Environmental: p = 0.553, and Oral: p = 0.235). Similarly, when OTUs were ranked as increasing or decreasing in each sample, relative to the untreated samples, the protocols did not have significantly different impacts (one-way ANOVA, Environmental: p = 0. 0.178 and Oral: p = 0.908). Despite non-significance, the published protocols generally performed better than the individual UV or NaClO treatments. In addition, some oral OTUs were reduced following each treatment, indicating that while decontamination is effective, it also impacts endogenous DNA.

#### Contaminant amplicon signals primarily originate from soil taxa

Genera absent in the HOMD but present in the samples (12 of 44) are potentially environmental. Eight of these genera are associated with soil microbiomes. The archaeal taxa, *Candidtus nitrososphaera*, is a common soil microorganism^65^, and bacteria from soil types expected in the archaeological context (i.e., irrigated agricultural soil, landfill, and freshwater sediments) were identified, namely *Pseudonocardia*, *Paludibacter*, *Paenisporosarcina*, *Pedomicrobium*, *Propionivibrio*, *Steroidobacter*, and *DA101*^66–74^. *Schwartzia* was also identified as an environmental contaminant and is found in ruminants, particularly cows^75^. Several taxa are known to be present in both environmental and human microbiota and include *SHD-231* (found in ruminants and human periodontal pockets)^76^ and *Hydrogenphaga* (found in the water flea (Daphnia) gut and human disease)^77,78^ and therefore may be endogenous or contaminant taxa. The final genus, *TG5*, is not found in the HOMD, but has previously been reported in the human mouth^79^, indicating disparity between methods of classifying oral taxa. Interestingly, microorganisms from non-oral human body sites were not detected, suggesting minimal contamination while handling. Together, this suggests that soil is the likely source of contaminant DNA within these ancient calculus samples, as all non-oral genera are typically isolated from soils and sediments. However, future should consider assessing how other contributing factors, such as length of storage and storage environment, impact contamination.

#### Amplicon taxa profile of ethanol washes

DNA within the ethanol washes was sequenced to identify the taxa that were released from the calculus samples during the decontamination process. We assessed the potential of gaining insight into the environmental information preserved on the outer surface of calculus by examining the taxa present within the ethanol washes. Ethanol washes contained low diversity and had a limited number of reads, as expected^31^. Not all samples could be successfully sequenced, resulting in the loss of two ethanol wash samples from the group treated with EDTA (Fig. 2B and C). In total, 77 genus-level OTUs were identified within the ethanol washes across all sample groups, and 59 of these were classified as environmental taxa (*i.e.,* not present in HOMD database). Of the 10.2 genera observed on average within the untreated samples, nearly half (47%) were environmental (4.8 genera). The largest proportion of environmental taxa was observed in the EDTA treatment group (n = 3); eight of ten genera in ethanol washes following EDTA treatment were environmental. The ethanol washes following the UV + NaClO treatment had fewer total genera than the untreated samples, and 33% were environmental (2.4 of 7.2 OTUs). Ethanol washes from UV treated samples contained more environmental genera than the untreated (51%; 6.4 of the 12.6 genera present), and NaClO-treated samples with an ethanol wash had 4.7 taxa, the lowest average of all groups (2.5 environmental genera (53.2%)). While only limited, stochastic taxa could be recovered from these ethanol washes, these findings support the idea that amplifiable DNA, largely originating from environmental microbes, is being washed away from the dental calculus surface following decontamination procedures.

### Shotgun data analysis

#### Robust ancient calculus signal obtained from ancient calculus

We explored the impacts of decontamination methods using shotgun metagenomic sequencing, as 16S rRNA approaches have been shown to have systemic biases for ancient dental calculus specimens during community analysis^64^. We first examined the general composition of taxa present in the sample to ensure a robust ancient calculus signal had been obtained. The dominant phyla (pre-filtering) across samples were Actinobacteria (35.0%), Proteobacteria (23.8%), and Firmicutes (17.0%). The predominant microbial species across all samples are typically found in the mouth, and include *Olsenella sp.* oral taxon 807 (13.9%), *Actinomyces sp. o*ral taxon 414 (11.8%), *Anaerolineaceae bacterium* oral taxon 439 (8.58%), *Fretibacterium fastidiosum* (5.56%), and *Desulfomicrobium orale* (5.50%)*.* The filtering process identified 53 species (22.6% of the total) that were found in both the samples and EBC (Supplemental Information). We removed all of these species from our dataset for the downstream analyses. None of these species are found in the HOMD database and most have been associated with non-oral microbiomes^80–84^. After EBC filtering, a total of 182 species were left in the shotgun dataset (Supplemental Information).

#### Decontamination protocols increase yields of oral microbes

**Figure 5:**
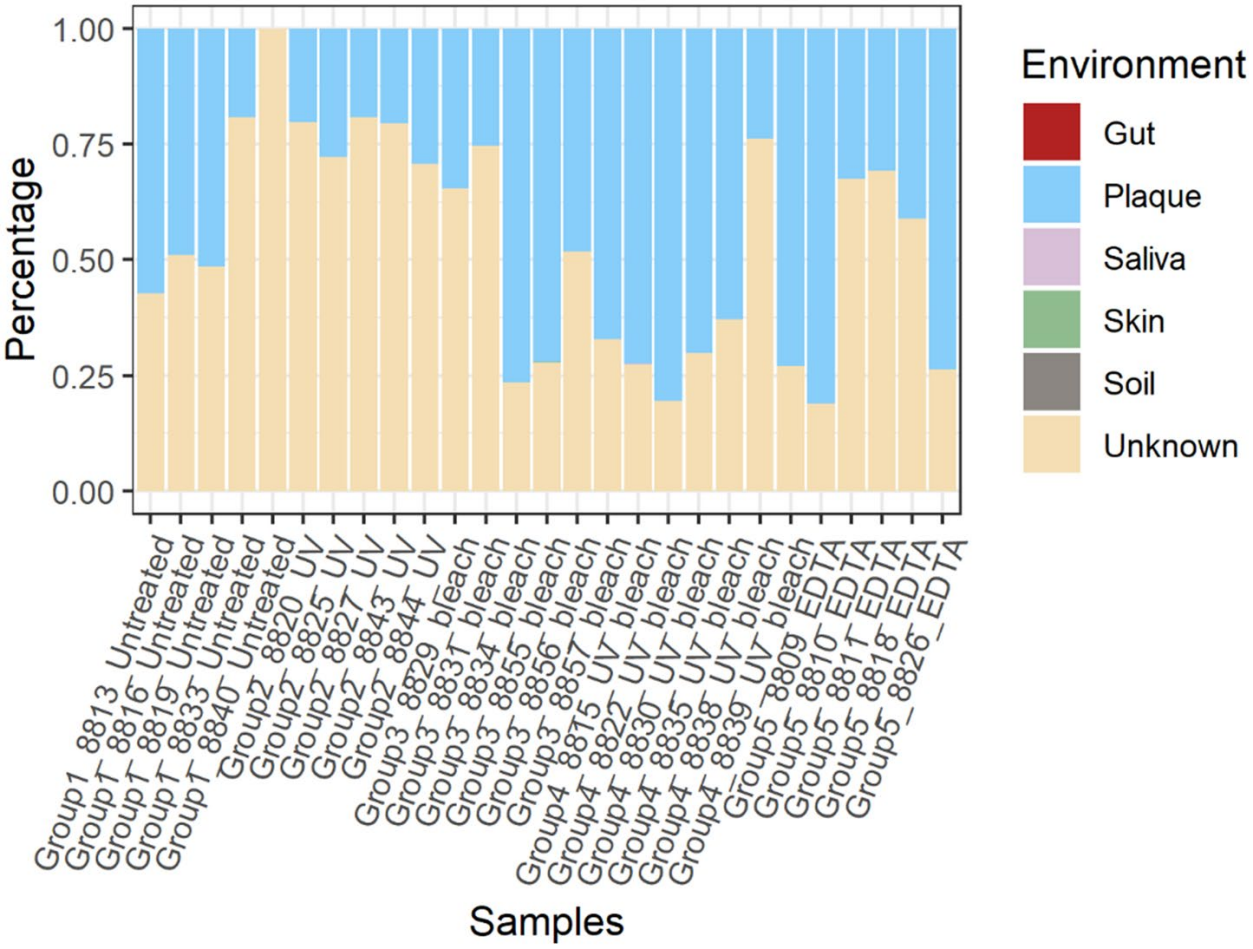
Stacked bar plots of Bayesian SourceTracker results for the shotgun dataset. The estimated proportions of source contribution at the species level, using gut, plaque, saliva, skin, and soil sources. Reads which could not be assigned to an environment were classified as ‘Unknown’ in SourceTracker. Plot was generated with ggplot2^60^.

SourceTracker was used to predict the mixing proportions of environmental contaminants and endogenous content in the shotgun metagenomic dataset (Fig. 5). SourceTracker results identified differences in the proportion of oral species obtained after the decontamination protocols. The UV + NaClO treatment group yielded the highest average of oral species (63.8%), followed by the sodium hypochlorite-only (54.0%) and EDTA (51.8%) treatments. An unexpected finding was that the UV-only group yielded a lower oral microbiome signature (23.4%) than the untreated group (35.3%). The proportions for unknown were considerably lower for the UV + NaClO (36.2%), NaClO (46.3%), and EDTA (48.1%) groups than the untreated (64.7%) and UV-only (76.6%) groups, suggesting that the three former decontamination procedures are more effective in increasing the chances of recovering oral taxa. Therefore, soil and skin contamination are more difficult to detect in this shotgun dataset compared to the 16S rRNA amplicon data. Nevertheless, the results from both analyses show a general trend that UV + NaClO, EDTA, and NaClO protocols increase the proportions of an oral signal from an archaeological dental calculus sample.

#### Decontamination protocols do not significantly impact shotgun metagenomic diversity of ancient dental calculus

To evaluate whether current decontamination protocols alter microbial diversity, we compare the alpha diversity (observed species and Shannon’s diversity index) of untreated samples to those treated with each decontamination method. There were no significant differences in diversity amongst the decontamination groups (Kruskal–Wallis; all p-values > 0.05). However, every decontamination method resulted in a higher mean of observed species than the untreated groups. The EDTA group had the largest variation among samples, although this was also non-significant from other groups (p-value = 0.0163, H = 6.528).

**Figure 6:**
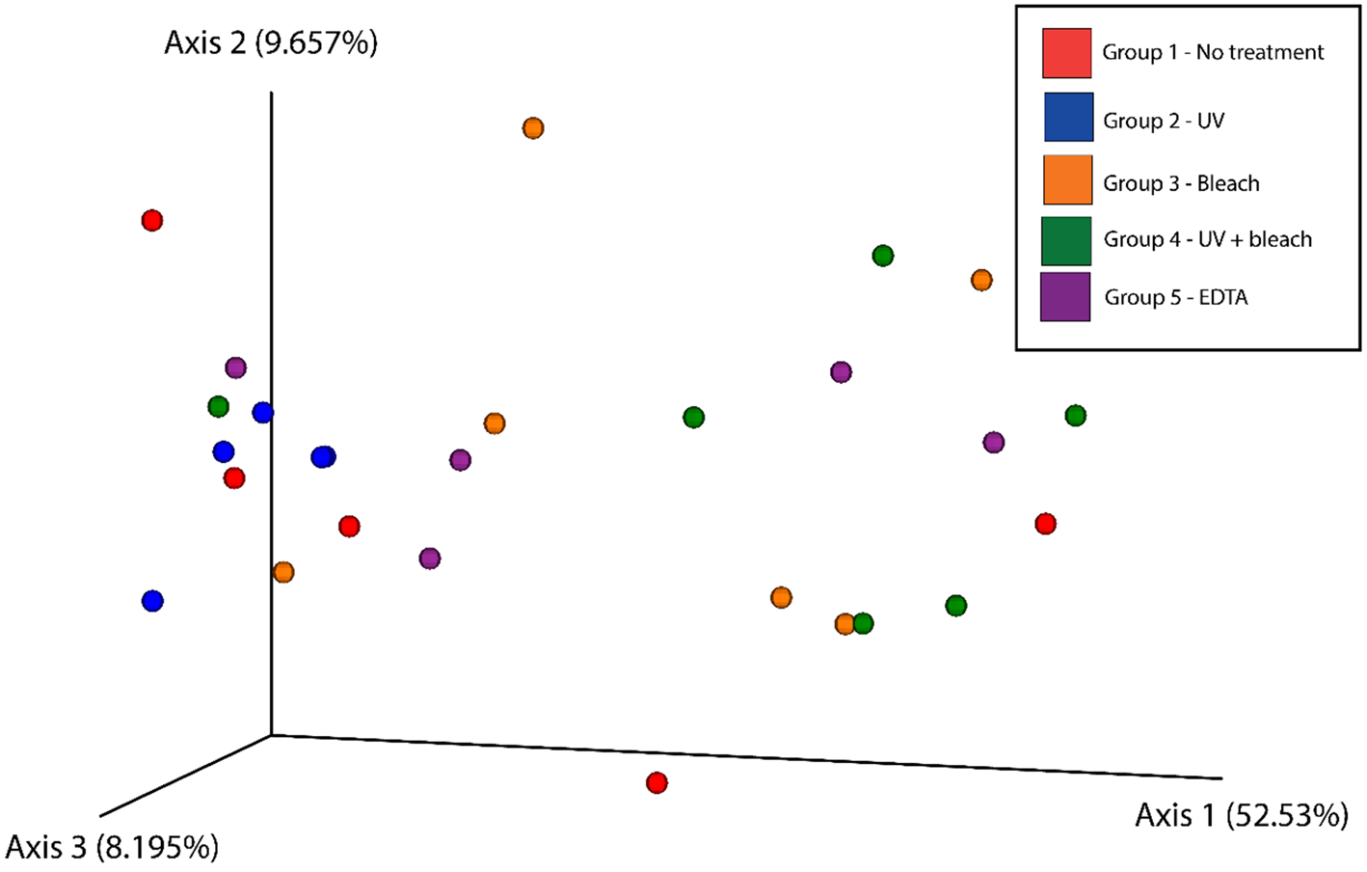
Principal Coordinates Analysis (PCoA) plot based on Bray–Curtis dissimilarity for the shotgun dataset. Points in three-dimensional space represent samples, each colored according to treatment. Statistical analysis revealed a non-significant separation between samples based on treatment (Kruskal–Wallis, *p* value > 0.05). Plot generated using the EMPEROR tool in QIIME2^48^.

Similarly, decontamination protocols did not significantly alter microbial composition within dental calculus. A PCoA analysis based on Bray–Curtis dissimilarity did not reveal clustering according to decontamination method (Fig. 6). Beta diversity metrics support this finding, as there were no significant differences between the decontamination protocols (PERMANOVA, t = 1.80386, p = 0.073). Overall, this finding suggests that decontamination does not significantly impact the total composition of ancient calculus samples during shotgun metagenomic analysis.

#### Decontamination protocols impacts the recovery of oral-associated species

A Kruskal Wallis test on species abundances and a G-test (Supplemental Information) for the presence and absence of species were performed to examine if specific species were impacted by decontamination. The Kruskal Wallis test did not identify any differentially abundant species in the untreated compared to treated groups (p > 0.05). In the G-test, eight environmental species were present in the untreated group but absent in all of the decontamination groups*,* including *Methanobrevibacter ruminantium, Mobiluncus curtisii, Ruminococcus flavefaciens, Comamonas serinivorans,* and *Rhodanobacter sp. Soil 772*. Additional environmental species were also present in the UV-treated only group, but absent in the EDTA and UV + NaClO methods, including *Serpentinomonas mccroryi, Cloacibacillus evryensis,* and *Schaalia turciensis*. In contrast, several oral-associated species were absent in the untreated group compared to the treated ones, including *Lachnoanaerobaculum saburreum* and *Selenomonas noxia*. A few species were also absent in both the untreated and UV-only groups, but present in the other three decontamination methods (*e.g., Streptococcus sanguinis, Rothia aeria, Leptotrichia sp.* oral taxon 212, *Streptococcus oralis,* and *Neisseria sp.* oral taxon 014). Three oral species—*Actinomyces* sp. oral taxon 414 (test-statistics: 372,696.5, p = 0.0), *Olsenella* sp. oral taxon 807 (test-statistic: 273,435.6, p = 0.0) and *Streptococcus sanguinis* (test-statistic: 429,185.8, p = 0.0)*—*were also more likely to be present in the EDTA and UV + NaClO groups than the others. In summary, these results support the argument that the UV + NaClO and EDTA protocols are more effective in decontamination than the other treatments.

## Discussion

Our results indicate that the UV + NaClO and EDTA treatments are effective in decontaminating archaeological dental calculus. Each treatment reduces the yield of environmental contaminant taxa, while increasing the yield of oral taxa in both amplicon and shotgun metagenomic approaches. Our analyses also support previous findings that amplicon sequencing is more sensitive to rare taxa, especially low-level contaminant taxa, than shotgun sequencing. These findings also support the idea that a combined UV radiation and NaClO submersion or a pre-digestion in EDTA are more effective decontamination methods than a single treatment with either UV radiation or NaClO.

### UV + NaClO and EDTA treatments are effective decontamination methods

Each decontamination method resulted in fewer identifiable contaminant taxa in comparison to untreated samples based on both amplicon and shotgun methodologies. However, removal of environmental taxa and increases in oral species were only observed in the EDTA and UV + NaClO decontamination approaches. In our amplicon dataset, both the EDTA treatment and UV and sodium hypochlorite treatment showed a reduction in specific environmental taxa, an increase in oral species, and a decrease in alpha diversity. Shotgun data confirmed an increase in oral associated taxa with both methods, although the removal of rare environmental species was less pronounced with this approach. While slight improvements in the removal of soil OTUs were observed with the UV + NaClO approach over the EDTA one, the stochasticity of results prevents us from discerning which method is more effective at removing environmental contamination. Consequently, both treatments appear to be effective at removing soil contaminant DNA from calculus samples. However, further studies exploring more samples, and decontamination analysis on samples from the same individual, should be performed to support this finding.

### The UV and sodium hypochlorite treatments alone are not as effective as the combined UV + NaClO treatment

While the UV-only and NaClO-only treatments decrease the number of contaminant taxa, the SourceTracker and G-test results in the amplicon analysis indicate that both were not as effective in decontaminating samples as the UV + NaClO and EDTA treatments. The limited effectiveness of UV treatment may be due to its inability to irradiate pockets, pits, and fissures in the rugose calculus surface. These pockets may shield contaminant microbes and their DNA from the UV radiation. The NaClO treatment also failed to reduce environmental contamination and resulted in an additional increase the total OTU count. However, many of these OTUs were novel, suggesting that the NaClO treatment resulted in the creation of non-biological DNA sequences via oxidative action. Low concentration of NaClO can cause base modifications which could have impacted the downstream taxonomic profiling^27^. These results suggest that despite the use of NaClO as a common practice in ancient DNA decontamination protocols, it may be creating a false-positive signal by shortening and damaging the DNA of exogenous microbes within a calculus sample. Previously studies have shown that UV is less effective than sodium hypochlorite during decontamination of short (> 700 bp) DNA fragments; however, our study suggests that either used alone are ineffective decontamination methods^85^. Critically, the combination of UV radiation and sodium hypochlorite immersion resulted in a decrease in environmental taxa and did not result in an increase in novel OTUs. A possible explanation for this is that the UV radiation first crosslinks contaminant DNA and the following sodium hypochlorite breaks it apart, which, in turn, prevents it from being amplified in downstream processes^85^. Further studies should consider investigating the effects of both UV and different concentrations and immersion times of NaClO, including concentrations and immersion times to explore their use for the decontamination of ancient microbiome samples^28^.

### Soil sediments are the main sources of contaminant OTUs

Environmental contaminant DNA can originate from a wide variety of sources, including soil, water, plant matter, decomposition of the body, archaeologists and museum curators, archaeologist tools, museum dust, etc. Our analysis identified that soil is the major source of contaminating DNA in calculus samples. Of the 12 environmental genera removed by all decontamination protocols, we identify eight of them from known soil or sediment sources. SourceTracker analysis of the amplicon dataset specifically attributed the contaminant DNA within calculus samples to microorganisms within parkland and agriculture soils, which is consistent with the urban gravesites where these samples were recovered. However, the microbial communities within soil can vary depending on geographic location^86^. Therefore, sediment from the archaeological site should be collected, when and if available, as it will provide the best comparison of contaminant DNA, rather than relying on soil microbial databases. If soil is not available from the site, then comparisons to these databases serve as the next best option to examine these effects. It is likely that this approach would allow for better identification of low-level environmental species during shotgun metagenomic analysis, which identified only a handful of environmental microorganisms compared to the amplicon methodology. Improved diversity and quality of environmental species reference genomes would likely improve our ability to detect soil and environmental contaminants in ancient dental calculus data sets.

Surprisingly, for the 16S and shotgun analysis, very few skin OTUs (< 1.0%) were observed in any of the samples, regardless of decontamination protocol or sequencing method. This outcome likely reflects minimal handling during excavation and curation at this specific site, as skin microbiota have previously been reported in ancient calculus^87^. The site used in this study is a primary example of rescue archaeology, where limited time was available to complete the survey and minimal sample handling occurred^29^. Further, the samples analyzed in this study were not housed in a museum collection, which also likely limited their exposure to human skin. These specific circumstances may have limited exposure to human skin microorganisms, but future studies should continue to monitor for skin contaminant OTUs or unique taxa that could be introduced through additional sample exposure after human remains are unearthed.

### Environmental contamination is easier to detect using 16S amplicon sequencing is than shotgun sequencing

We were only able to perform genus-level analyses with the amplicon data (16S rRNA sequences), which may have masked unique environmental contaminant DNA^88^. For example, *Actinomyces* and *Streptococcus* are genera considered part of the oral bacterial community and are included in HOMD. However, both genera contain multiple species that are commonly found in the environment^89,90^ Consequently, 14 of the known 47 *Actinomyces* species are not included on HOMD, as are 73 of 116 known *Streptococcus* species. Therefore, the signal from environmental species cannot be detected and removed from the oral signal with genus-level identifications. Inference of biologically or culturally relevant patterns can then result from incorrect abundance measures. To overcome some of these biases, we also performed shotgun sequencing on dental calculus samples. This approach is preferable, as it provides the ability to obtain detailed species and strain information, affords a greater resolution of the microbial community within the sample^63^, and gives insights on the functional capacity. However, this method did not identify as many environmental contaminants, which were likely present in low levels based on the amplicon sequencing data. The absence of such species is likely a technical artifact of shotgun sequencing. The shotgun analysis did identify the presence of environmental species, such as *Proteiniphilum acetatigenes, Proteiniphilum saccharofermentans,* and *Petrimonas mucosa,* in the untreated and UV-treated groups while absent in the NaClO, UV + NaClO, and EDTA groups, suggesting that specific species tracking of contaminants is tenable with shotgun metagenomic sequencing. Improvements in reference sequences and analytical tools will undoubtedly improve our ability to track contaminant species in shotgun metagenomic studies moving forward. Future research should investigate whether the microbes removed during decontamination are linked to the burial environment (*e.g.* soil acidity and climate). Because environmental taxa will vary across archaeological contexts, it is important to investigate the impacts decontamination protocols have in a wide range of archaeological settings.

### Limitations of this study

This study has several limitations. For instance, we used dental calculus samples from different individuals, so we exclude the possibility that the microbial profile differences among the groups are due to biological processes rather than the decontamination protocols. Ideally, it would make the most sense to apply each treatment to an individual dental calculus sample and compare the observed differences. This approach was not possible due to the size of the calculus fragments. Alternatively, one could apply each treatment to different teeth from a single individual. However, such effort is also limited, as the composition of oral microbiota varies according to tooth type (*e.g.,* canine vs molar) and tooth surface (*e.g.,* buccal vs lingual)^2,91^. Another limitation is that these samples had minimal handling during the time of excavation and were all buried in the same geographic area, which is not necessarily the case for other archaeological assemblages. Samples that have been handled with less precautions may provide different insights into the efficacy of decontamination protocols. Future testing should also examine the efficacy of the most appropriate decontamination protocols according to specific burial environments. For instance, different types of a soils or soils with different chemical compositions may make the UV + NaClO treatment less effective than the EDTA treatment and vice versa. A way to potentially address some of these concerns is to set up an in vitro modeling study design involving a controlled setting with soil and skin contamination. While this approach would not address taphonomic concerns, it could overcome the limitations imposed by the amount of calculus per sample. In summary, these findings add to the growing body of evidence that decontamination protocols impact downstream analyses, and therefore, the field should devote more resources into finding the most robust approach.

## Conclusion

Contaminant DNA is a major concern for paleomicrobiological studies. The lack of a universal decontamination protocol for the field makes it difficult to compare study findings across groups, especially when merging microbial relative abundances from multiple analyses. Our results suggest that the UV + NaClO and EDTA treatments are the most effective since they increase the sequencing depth of oral taxa while also remove up to 9.1% of environmental contaminants within a sample. Such findings also indicate that the two likely improve downstream analyses, such as dimension reduction, visualization, clustering, and differential abundance analysis. Moving forward, we recommend that scholars wear gloves and face coverings when sampling for calculus either in the field or a museum, collect at least one soil sample from the field that will be sequenced, process samples in labs committed to only performing aDNA research, decontaminate samples with either the UV + NaClO or EDTA method, sequence at least one DNA extraction control along with the collected soil sample, and publish sequencing data for both the soil and laboratory controls. Because the paleomicobiological field is unable to resample from irreplaceable biomaterials, it should strive towards standardizing protocols from collecting samples through sequencing. Such measures will improve the overall quality of ancient sequencing data. Basic laboratory practices of decontaminating ancient samples is a key element of acquiring high quality data.

## Supplementary Information

Supplementary Files.

## Data Availability

All raw sequence data from this study is available at NCBI SRA (https://www.ncbi.nlm.nih.gov/sra) under project number: PRJNA688065. All R scripts can be found at https://github.com/SterlingLWright/aDNA_Decontam_protocols.
